# Assessment of Long-Term Watershed Management on Reservoir Phosphorus Concentrations and Export Fluxes

**DOI:** 10.3390/ijerph15102169

**Published:** 2018-10-02

**Authors:** Xiaolin Huang, Han Chen, Fang Xia, Zhenfeng Wang, Kun Mei, Xu Shang, Yuanyuan Liu, Randy A. Dahlgren, Minghua Zhang, Hong Huang

**Affiliations:** 1 Zhejiang Mariculture Research Institute, Wenzhou 325035, China; xiaolinnlh@hotmail.com; 2 School of Public Health and Management, Wenzhou Medical University, Wenzhou 325035, China; chenh@iwaterlab.com (H.C.); xiafang@iwaterlab.com (F.X.); wangzf@iwaterlab.com (Z.W.); meikun@iwaterlab.com (K.M.); copepod@sina.com.cn (X.S.); liuyuan@iwaterlab.com (Y.L.); 3 Southern Zhejiang Water Research Institute (iWATER), Wenzhou Medical University, Wenzhou 325035, China; radahlgren@ucdavis.edu (R.A.D.); mhzhang@ucdavis.edu (M.Z.); 4 Key Laboratory of Watershed Environmental Science and Health of Zhejiang Province, Wenzhou 325035, China; 5 Department of Land, Air, and Water Resources, University of California, Davis, CA 95616, USA

**Keywords:** phosphorus, concentration, reservoir, anthropogenic sources, watershed management

## Abstract

Source water nutrient management to prevent eutrophication requires critical strategies to reduce watershed phosphorus (P) loadings. Shanxi Drinking-Water Source Area (SDWSA) in eastern China experienced severe water quality deterioration before 2010, but showed considerable improvement following application of several watershed management actions to reduce P. This paper assessed the changes in total phosphorus (TP) concentrations and fluxes at the SDWSA outlet relative to watershed anthropogenic P sources during 2005–2016. Overall anthropogenic P inputs decreased by 21.5% over the study period. Domestic sewage, livestock, and fertilizer accounted for (mean ± SD) 18.4 ± 0.6%, 30.1 ± 1.9%, and 51.5 ± 1.5% of total anthropogenic P inputs during 2005–2010, compared to 24.3 ± 2.7%, 8.8 ± 10.7%, and 66.9 ± 8.0% for the 2011–2016 period, respectively. Annual average TP concentrations in SDWSA decreased from 0.041 ± 0.019 mg/L in 2009 to 0.025 ± 0.013 mg/L in 2016, a total decrease of 38.2%. Annual P flux exported from SDWSA decreased from 0.46 ± 0.04 kg P/(ha·a) in 2010 to 0.25 ± 0.02 kg P/(ha·a) in 2016, a decrease of 44.9%. The success in reducing TP concentrations was mainly due to the development of domestic sewage/refuse collection/treatment and improved livestock management. These P management practices have prevented harmful algal blooms, providing for safe drinking water.

## 1. Introduction

Anthropogenic P use has increased dramatically in the past century due to development of industry, agriculture, and animal husbandry, as well as increasing population and living standards [1,2]. For instance, total chemical P fertilizer usage in China increased from 273.3 × 10^4^ ton P/a in 1980 to 690.5 × 10^4^ ton P/a in 2000 and 843.1 × 10^4^ ton P/a in 2015, total increases of 153% and 209%, respectively [3]. Across mainland China, net anthropogenic phosphorus inputs (NAPI) for 1981, 1990, 2000, and 2009 were 1.90, 2.95, 4.15, and 4.65 kg P/(ha·a), respectively [4]. Notably, the development of waste treatment and P management has lagged increased P use resulting in excessive P inputs to the environment. As a result, excessive P concentrations/fluxes are a major cause of eutrophication and harmful algal blooms in lakes and reservoirs in China, as well as worldwide [5,6,7].

Reducing P losses to the environment are essential to avoid further eutrophication and allow recovery in lakes and reservoirs [8]. With regard to drinking water supplies, water may become unusable due to harmful algal blooms [9]. Hence, many watershed pollution control plans were developed to enhance watershed P management and reduce P concentrations and loads to reservoirs and other freshwater ecosystems [10,11]. P reduction strategies have been successful in some watersheds, yet many other watersheds have not responded as expected, even after decades of intensive efforts [12,13,14]. To guide future environmental management, both successful and unsuccessful outcomes must be rigorously analyzed to inform future management options.

Excessive P concentrations and loads in reservoirs are largely attributed to the overuse of anthropogenic P (e.g., industrial and domestic sewage, P fertilizer, livestock excrement) within the watershed. Thus, improvement of reservoir water quality depends on pollution load reductions by means of integrated watershed pollution control measures including source management [15], process control [16], and end-of-pipe treatment [17]. It has been generally recognized that source management is a fundamental strategy for sustainable watershed P management and integrated pollution control [15]. However, different anthropogenic P sources lead to different fate and transport dynamics within watersheds resulting in contrasting impacts on downstream reservoirs [18,19]. For instance, industrial sewage (i.e., point source) without treatment often has an immediate and direct impact on receiving waters while P fertilizer (non-point source) transport may be considerably lagged (by decades) due to transient storage from phosphate-adsorption reactions with soil minerals [20,21,22]. In general, concentrations of major water quality constituents represent the water quality condition of receiving waters, while source strengths (i.e., loads or fluxes) of pollutants represent the watershed environmental pollution stress. Hence, understanding long-term changes in reservoir P concentrations/fluxes and their relationship with watershed anthropogenic P source inputs is critical for devising effective watershed management plans to reduce P loads to receiving waters. Therefore, it is informative to follow the response of reservoir P concentrations/fluxes to reductions in watershed-scale anthropogenic P inputs over time to evaluate the effectiveness of various watershed P management strategies and how the reservoir responds to dynamic P concentrations.

The Shanxi Drinking-Water Source Area is located in Zhejiang Province, China and provides water for ~7 million people. This area experienced severe water quality impairment from P inputs before 2010, with a subsequent improvement resulting from comprehensive environmental regulations and implementation of nutrient reduction measures since 2010 [23,24,25]. This paper analyzed the long-term (12 year) record of reservoir total phosphorus (TP) concentrations/fluxes and watershed anthropogenic P input loads (e.g., domestic sewage, livestock, and fertilizer) to determine the influence of different anthropogenic P sources on reservoir TP concentrations, assess the performance of the watershed management practices, and provide recommendations for future watershed P management strategies. This analysis provides a scientific basis to guide watershed P management for drinking-water source areas that is applicable to nutrient management and remediation strategies for watersheds worldwide.

## 2. Materials and Methods

### 2.1. Study Area and Data Collection

**Figure 1:**
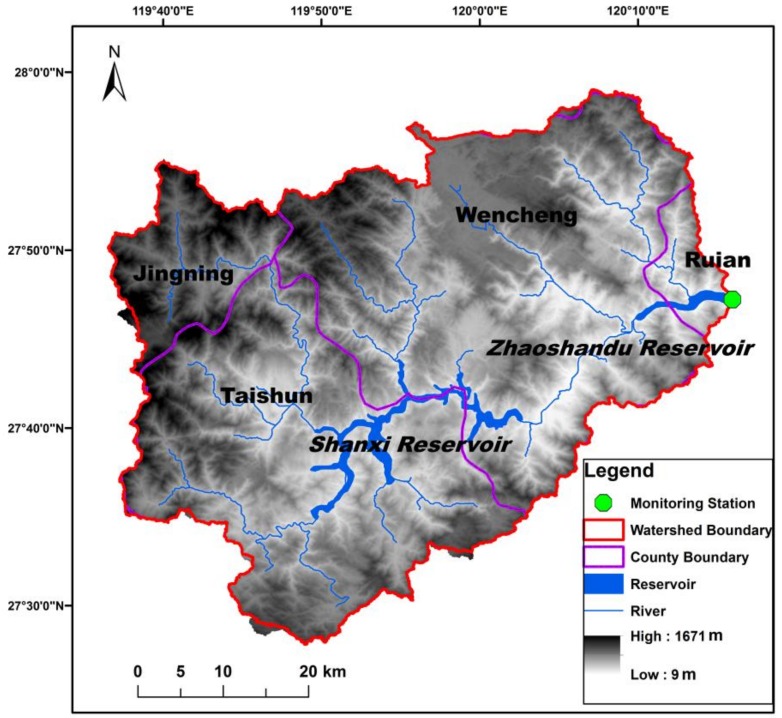
Geographic location and water quality monitoring site location.

Shanxi Drinking-Water Source Area (SDWSA) is located in the headwaters of the Feiyun River Watershed in Zhejiang Province, China (Figure 1). Total watershed area is 2303 km^2^ and falls within four administrative counties (Jingning, Taishun, Wencheng, and Ruian). SDWSA is the major drinking water source for ~7 million people and consists of a multi-annual regulating reservoir (Shanxi Reservoir, storage capacity of 1.8 × 10^9^ m^3^) with a water diversion project (Zhaoshandu Reservoir, storage capacity of 3.4 × 10^7^ m^3^) located 30 km downstream of Shanxi Reservoir. The watershed has a subtropical monsoon climate with mean annual precipitation of 1870 mm and temperature of 17 °C [24]. Mean watershed elevation is 573 m (9–1671 m) with land use dominated by forest (75%), agriculture (15%), and developed land (8%). The watershed is dominated by highly weathered, iron oxide-rich red soils corresponding to Ultisols/Oxisols in U.S. Soil Taxonomy [25]. Current population density within the watershed is 215 people/km^2^, which is about 1.5 times the national average (143 people/km^2^) [23].

**Figure 2:**
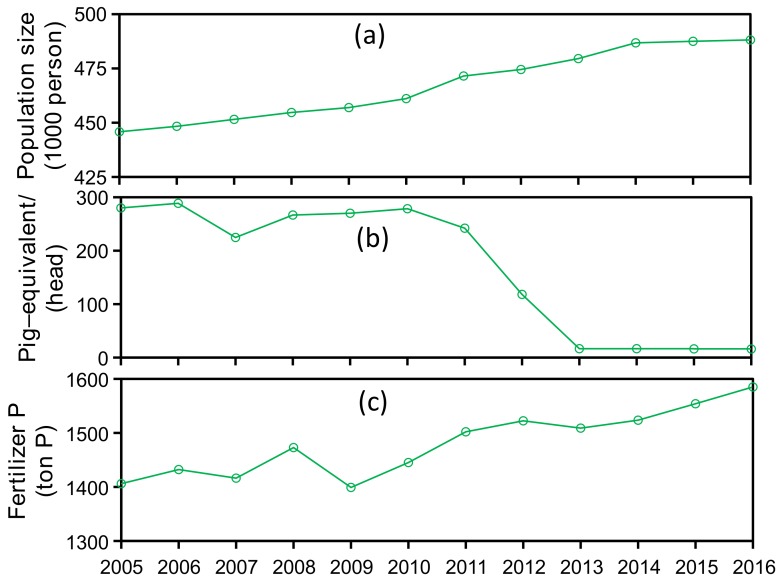
Annual (**a**) population size, (**b**) livestock production, and (**c**) P fertilizer application amount within Shanxi Drinking-Water Source Area (SDWSA). Livestock production is transformed to pig-equivalent values (1 pig equals 45 poultry, 30 rabbits, 3 sheep, and 0.2 cattle, respectively) according to Discharge Standard of Pollutants for Livestock and Poultry Breeding (GB18596-2001).

Cyanobacteria blooms occur in some backwater regions of the reservoirs and TP is considered the key water quality parameter contributing to eutrophication and harmful algal blooms [23]. TP in Zhaoshandu Reservoir was monitored by the local Water Resources Bureau on an approximately monthly basis during 2005–2016 (*n* = 143) (Figure 2). Annual reservoir release volumes from Zhaoshandu Reservoir were obtained from the local Water Resources Bureau. TP was measured using the ammonium molybdate spectrophotometry method with a detection limit of 0.01 mg/L (GB11893-89) [26].

### 2.2. Anthropogenic P Calculation

Annual anthropogenic P inputs to SDWSA from domestic sewage, livestock waste, and fertilizer were calculated for a 12-year period (2005–2016):

Anthropogenic P = Sewage P + Livestock P + Fertilizer P(1)

Domestic sewage P is the sum of human excretion and P-containing detergents, and the annual sewage P was calculated as the population multiplied by the emission rate. Livestock waste P is the sum of excretions from major livestock and poultry types and was calculated as the annual livestock breeding number multiplied by the excretion rate. The population size and the breeding numbers for livestock and poultry in each administrative county were derived from statistical yearbooks and interview surveys (Figure 2). Emission parameters for humans and livestock are listed in Table 1 [1]. Fertilizer P is mainly sourced from phosphate fertilizer and animal waste applications and was derived from statistical yearbooks and field investigations for each administrative county. It should be noted that atmospheric P deposition is an anthropogenic P input, yet it was not included in this paper due to the lack of available data for SDWSA. A previous study estimated atmospheric P deposition in Zhejiang Province of ~5% of total watershed anthropogenic P inputs [27] and therefore it is believed to be minor component of P inputs.

Anthropogenic P sources in the watershed were calculated by overlaying land-use types and human and livestock populations on the river basin boundary and administrative boundary GIS layers. Data for population size, livestock breeding numbers, and the estimated emission parameters for humans and livestock inevitably have inherent errors when calculated at the large watershed scale. To assess uncertainty, a total of 1000 Monte Carlo simulations were performed to obtain the means and 95% confidence intervals for anthropogenic P sources. All data (Figure 2) used in anthropogenic P source estimations were assumed to follow a uniform distribution (original data × 90%, original data × 110%), as well as the parameters in Table 1 (estimated value × 90%, estimated value × 110%).

### 2.3. Phosphorus Export Flux Estimation

The area-based, annual TP flux exported from Zhaoshandu Reservoir was estimated from annual runoff volume, annual average TP concentration, and watershed area.

Flux = Runoff × Concentration/Area(2)

where, Flux represents annual P flux (kg P/(ha·a)), Runoff represents annual runoff volume (1000 m^3^), Concentration is the annual average TP concentration (mg/L), and Area is the watershed area (ha). All annual average TP concentrations were assumed to follow a uniform distribution (average × 90%, average × 110%). A total of 1000 Monte Carlo simulations were performed to obtain the mean and 95% confidence interval for annual P fluxes.

## 3. Results and Discussion

### 3.1. Long-Term Changes of Reservoir TP Concentration and Flux

**Figure 3:**
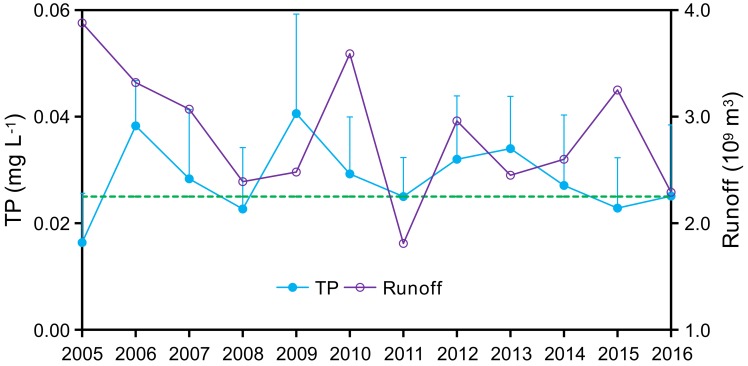
Annual runoff volume and annual average total phosphorus (TP) concentrations in Zhaoshandu Reservoir (2005–2016). The vertical error lines are the standard deviation of TP, and the horizontal dotted line represents the Grade II water quality standard goal for TP (0.025 mg/L).

Mean annual TP concentrations in Zhaoshandu Reservoir ranged between (mean ± SD) 0.017 ± 0.009 and 0.041 ± 0.019 mg/L for the 2005–2016 study period (Figure 3). Being a drinking-water source area, the water quality mandate for Zhaoshandu Reservoir is to maintain Grade II or better conditions (TP ≤ 0.025 mg/L) according to Chinese Water Quality Standards (GB3838-2002) (Table 2). TP concentrations fluctuated among years and several monthly TP concentrations exceeded the Grade II TP standard of 0.025 mg/L. Cyanobacteria blooms were documented in several backwater regions of Shanxi Reservoir and in the upstream portion of Zhaoshandu Reservoir in 2008 and 2009 leading to implementation of comprehensive environmental regulations by the local government after 2010 [23]. These efforts included implementation of improved domestic sewage collection and treatment, residential refuse collection, livestock waste management, and restoration of major tributaries [24,25].

**Figure 4:**
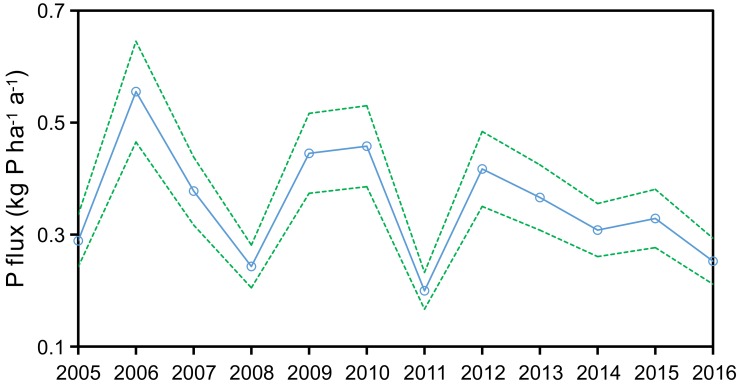
Annual P flux exported from Zhaoshandu Reservoir during 2005–2016. Dotted lines denote the 95% confidence interval obtained from Monte Carlo simulation.

For the post-best management practices (BMPs) implementation period, annual average TP concentrations decreased from 0.041 ± 0.019 mg/L in 2009 to 0.025 ± 0.013/mg L in 2016, a decrease of 38.2% (Figure 3). Meanwhile, annual P flux exported from Zhaoshandu Reservoir decreased from 0.46 ± 0.04 kg P/(ha·a) in 2010 to 0.25 ± 0.02 kg P/(ha·a) in 2016, a decrease of 44.9% (Figure 4). Importantly, TP concentrations have consistently met the Grade II TP water quality standard since 2015 and cyanobacteria blooms have not been detected, providing for safe drinking water [25]. These positive changes in water quality parameters in Zhaoshandu Reservoir after implementation of watershed scale nutrient BMPs in 2010 indicate that the BMPs were effective in improving reservoir water quality. However, reservoir TP concentrations consistently met Grade II TP water quality standards only since 2015 (Figure 3) indicating a time lag in the response of reservoir TP concentrations to watershed BMPs [13].

### 3.2. Long-Term Changes in Watershed Anthropogenic P Sources

**Figure 5:**
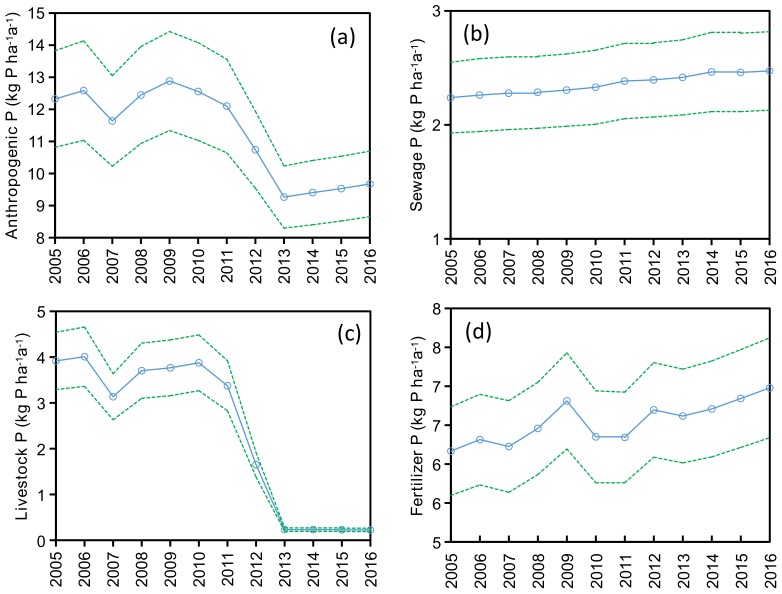
Annual (**a**) anthropogenic P, (**b**) sewage P, (**c**) livestock P, and (**d**) fertilizer P inputs within SDWSA (Shanxi Drinking-Water Source Area) during 2005–2016. Green shading denotes the 95% confidence interval obtained from Monte Carlo simulation.

The major anthropogenic P inputs to SDWSA were domestic sewage, livestock excrement, and phosphorus fertilizer. The annual P source inputs during 2005–2016 are shown in Figure 5. Inputs of P from livestock decreased appreciably after 2010, yet P inputs from domestic sewage and fertilizer both increased with time. Source strengths of P from sewage and fertilizer increased from 2.24 and 6.17 kg P/(ha·a) in 2005 to 2.47 and 6.98 kg P/(ha·a) in 2016, respectively, equivalent to increases of 10.4% and 13.2%, respectively. In contrast, the source strength of P from livestock waste decreased from 3.92 kg P/(ha·a) in 2005 to 0.23 kg P/(ha·a) in 2016, a decrease of 94.3%. Overall, the net changes in anthropogenic P inputs decreased from 12.3 to 9.7 kg P/(ha·a) over the 12-year study period.

**Figure 6:**
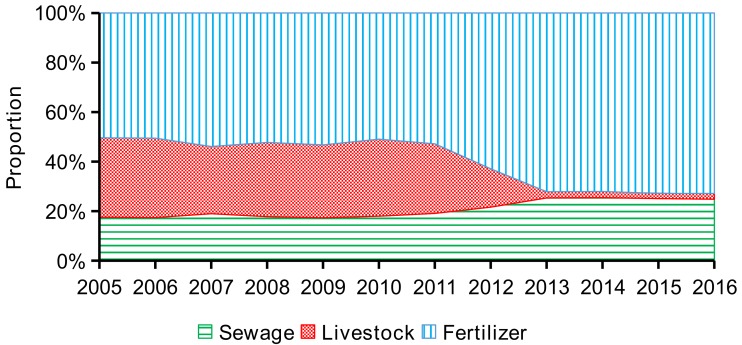
Source apportionment of anthropogenic P inputs within SDWSA (Shanxi Drinking-Water Source Area) (2005–2016).

For source apportionment, P from domestic sewage, livestock, and fertilizer accounted for 18.4 ± 0.6%, 30.1 ± 1.9%, and 51.5 ± 1.5% of total anthropogenic P inputs during 2005–2010 compared to 24.3 ± 2.7%, 8.8 ± 10.7%, and 66.9 ± 8.0% for the 2011–2016 period, respectively (Figure 6). Domestic sewage P sources mainly included human excretions and P-containing detergents [4], and therefore were predictably increased due to increasing population within the watershed (445,800 in 2005, 461,000 in 2010, and 488,100 in 2016). Animal waste treatment and reductions in livestock and poultry populations were aggressively promoted after 2010, with areas for livestock and poultry production either banned or subject to a maximum number of livestock (Figure 2). The increased use of P fertilizer over the study period resulted from the local government vigorously promoting agricultural crop production to compensate for the loss of the livestock and poultry industry. For instance, the economic value of agricultural production in Wencheng County more than doubled between 2010 and 2016 (4.87 billion to 9.84 billion RMB). The rapid increase in agricultural crops resulted in an overall increase in P fertilizer use. In spite of the large increase in P fertilizer use in SDWSA, overall anthropogenic P inputs decreased by 21.5% over the study period.

### 3.3. Response of Reservoir TP Concentration and Flux to Watershed Anthropogenic P Inputs

During 2005–2016, the percentages of watershed anthropogenic P exported in riverine TP fluxes were 1.7–4.4%, which are consistent with the range reported in previous studies (Table 3). Fertilizer P accounted for more than 65% of anthropogenic P inputs to SDWSA, and the increasing use of P fertilizer represents a considerable risk both today (runoff/erosion removal) and in the future as legacy P maybe remobilized.

Different pollution sources experience different fate and transport processes and therefore have differential effects on streams and reservoirs. Point source pollution (e.g., industrial and municipal waste water) can quickly influence water quality as it is directly discharged into surface waters [33]. In contrast, non-point source pollution (e.g., agricultural fertilizer, urban surface runoff) is not only determined by source strength, but also by transport processes along the terrestrial-aquatic hydrologic flow path [34]. Domestic sewage and refuse collection in this watershed was substantially enhanced since 2010 with municipal sewage and rural refuse collection reaching 98% and 100% in 2015, respectively. Municipal sewage was discharged after being treated and meeting effluent requirements, while the rural refuse was collected and transported to a landfill site for disposal. As a result, the final input of sewage and refuse P to SDWSA rapidly decreased in spite of increased human population.

P inputs from livestock to SDWSA mainly originate as waste water runoff from livestock and poultry production. Unlike scatter-feed operations used by small farm operations, large-scale, intensive livestock and poultry production creates large amounts of animal wastes within a small area. Thus, these large-scale animal operations act in many ways like point-source pollution, which immediately and directly influence the water quality of receiving waters. Therefore, the large reduction of livestock populations within SDWSA resulted in a rapid and obvious improvement on reducing TP concentrations in the reservoir since the large-scale and intensive livestock industry was prohibited in 2010 [25]. It should be noted that the response of TP concentration and flux to anthropogenic P source strengths may be attributable to many additional factors, including variable weather conditions (e.g., rainfall amounts, storm intensities), changing of land-use type, livestock scale and agricultural practices, as well as ‘legacy P’ dynamics [1,18,21].

### 3.4. Recommendation for Watershed P Management

Response of TP concentrations in Zhaoshandu Reservoir to changes in watershed anthropogenic P inputs indicated that BMPs reducing domestic and animal waste inputs were highly effective and showed rapid improvements in less than 5 years. However, water quality remediation is generally a difficult and long-term task, and more efforts are necessary to document existing achievements for potential application to drinking water security worldwide. From a practical point of view, insufficient financial resources and ecological/social compensation for changes in land use are difficult impediments to overcome at the large watershed scale. Government funding is necessary to ensure the construction, operation, and maintenance of domestic sewage treatment facilities. Economic development in many rural areas of developing countries is relatively slow due to the concentration of industrial development in urban centers. Hence, the participation of private enterprise is beneficial and should be encouraged, especially in rural areas that act as watersheds for drinking water reservoirs. Nowadays, collaborative partnerships such as Build-Operate-Transfer (BOT) and Public-Private-Partnerships (PPP) have been successfully applied in domestic sewage treatment projects and are showing great potential [35,36,37]. However, risk assessment and management must be considered when private enterprise is participating since failure may contribute to catastrophic damage to the environment [38,39]. River basin ecological compensation is becoming more common as an incentive to drive cutting edge pollution abatement in several countries and regions [40]. With respect to SDWSA, ecological compensation was not implemented to facilitate land-use change in the watershed. This study demonstrated that transformation of the livestock and breeding industry had a dramatic impact on TP reductions in SDWSA. Many livestock and poultry farmers in the watershed were asked to convert their livestock operations to environmentally friendly agriculture. However, many individuals lacked the necessary skills and capital to transform and manage such new enterprises. Therefore, transformation of the livestock industry to agricultural cropland faced great risks and required intensive assistance (e.g., capital and technical support, production marketing) from government and social–economic networks.

Watershed erosion from upland soils and also shoreline erosion associated with fluctuating reservoir water levels has the potential to contribute large TP inputs, as much of the non-point source P is associated with eroded sediments. It has been shown that sediment-associated P can be rapidly transformed to biologically available forms when the sediments become reduced in anoxic bottom waters of reservoirs or lakes [41,42,43]. SDWSA is dominated by subtropical red soils (Ultisols/Oxisols) occurring on steeply sloping topography with a mean watershed slope of 21.3% (0–68.8%). Forests were the predominant historic vegetation, but deforestation has occurred to support new agricultural enterprises. Today, forest cover in SDWSA is ~75% with a decreasing trend in recent years. Since this area is frequently hit by typhoons, catastrophic landslides have occurred, in part due to decreased hillslope strength as the roots from the deforested areas decay and no longer provide support for the hillslope [44]. To reduce soil erosion, a program to identify critical watershed areas for potential landslides and soil erosion is strongly warranted [45]. Areas with high potential for landslides and soil erosion should be considered for revegetation or appropriate BMP implementation to reduce erosion potential [46,47].

Impoundment of Shanxi Reservoir began in 1999 and a distinct zone of shoreline erosion has developed due to wave action associated with hydro-fluctuation over the past two decades. Shoreline erosion has contributed to the loss of vegetation due to soil loss, rockslides, and landslides. As a result, shoreline erosion has resulted in the complete loss of the soil profile to the depth of the underlying bedrock over several vertical meters (~10–15 m) associated with the fluctuating reservoir levels. Shoreline erosion is an unavoidable P source, yet effective and feasible controls to prevent or slow shoreline erosion are still lacking [48]. Thus, reservoir water quality is interactively influenced by anthropogenic P source inputs and erosion processes associated with both land-use change and shoreline erosion. Considering that erosion is strongly affected by natural conditions (e.g., topography, landform, soil, extreme weather events), it is often very difficult to address at the watershed scale. Thus, efforts that address anthropogenic P source inputs (e.g., domestic and livestock wastes and fertilizer applications) will have a more rapid response on TP concentrations in SDWSA reservoirs.

## 4. Conclusions

The changes in TP concentrations and fluxes from SDWSA relative to watershed anthropogenic P source inputs and watershed BMPs implementation during 2005–2016 were assessed. Overall anthropogenic P inputs decreased by 21.5% over the study period. Annual average TP concentrations in SDWSA decreased from 0.041 ± 0.019 mg/L in 2009 to 0.025 ± 0.013 mg/L in 2016, a total decrease of 38.2%. Annual P flux exported from SDWSA decreased from 0.46 ± 0.04 kg P/(ha·a) in 2010 to 0.25 ± 0.02 kg P/(ha·a) in 2016, a decrease of 44.9%. These positive changes in water quality parameters and the decrease in TP fluxes from the reservoir system indicate that the BMPs were effective in improving reservoir water quality. The success of watershed management practices in SDWSA was mainly due to development of domestic sewage treatment, reductions in livestock numbers, and enhanced livestock waste treatment which reduced overall anthropogenic P inputs by 21.5% during the study period. The increasing use of P fertilizer may hinder future reductions in reservoir P concentrations as legacy P pools are remobilized by runoff/erosion and leaching. Watershed management faces many challenges from practical difficulties, as well as difficulty in controlling natural factors. Efforts that address anthropogenic P source inputs, especially from point sources and livestock wastes, will achieve a more rapid response for reducing watershed P loads to downstream receiving waters.

## Figures and Tables

**Table 1:** Estimated emission parameters for humans and livestock [4].

Parameter	Human Excretion	Human Detergent	Pig Excretion
Estimated value	0.52 kg P/(person·a)	0.62 kg P/(person·a)	3.17 kg P/(animal·a)

**Table 2:** Chinese Water Quality Standards (GB3838-2002) for reservoir total phosphorus (TP) concentrations.

Grade	I	II	III	IV	V
TP (mg/L)	≤0.01	≤0.025	≤0.05	≤0.1	≤0.2

**Table 3:** Percentage of net anthropogenic P inputs exported in riverine TP fluxes in selected watersheds.

Percentage/%	Watershed	Reference
11.9–61.1	Guayas River, Ecuador	[28]
0.66–8.65	Chesapeake Bay, USA	[29]
3.6–5.4	Lake Michigan, USA	[1]
5.6–25.3	Lake Erie, USA	[1]
2.0–72.0	Central Valley, California, USA	[30]
1.6–14.2	Yongan River, China	[21]
1.5–19.2	Huai River, China	[18]
2.0–9.0	Hongze Lake, Zhejiang, China	[31]
2.3–7.9	Jingning Region, Hunan, China	[32]
